# Parallel pathways at the auditory periphery

**DOI:** 10.1186/1471-2202-15-S1-P193

**Published:** 2014-07-21

**Authors:** Marcos A Cantu

**Affiliations:** 1 Center for Computational Neuroscience and Neural Technology (CompNet), Boston University, Boston, MA, USA; 2 Graduate Program for Neuroscience (GPN), Boston University, Boston, MA, USA

## 

**Figure 1:**
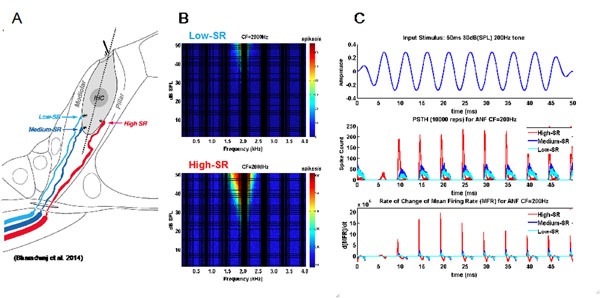
Innervation sites (A), tuning curves (B) and temporal response properties for Low- and High- SR ANFs

We should consider the possibility that Low- and High- spontaneous rate (SR) auditory nerve fibers (ANFs) [1] constitute two different parallel pathways at the auditory periphery. The present study used a computational model of the auditory periphery [2] to demonstrate that Low- and High- SR ANFs have contrasting response properties. Anatomical studies suggest that Low- and High- SR ANF types have separate innervation sites (Figure 1A) on the same inner hair cell; lower-SR fibers synapse on the modiolar side and high-SR fibers synapse on the pillar side [3]. My hypothesis, prior to modeling the tuning curves (Figure 1B), was that Low Spontaneous Rate (Low-SR) fibers have a higher threshold for simulation and thus will have demonstrably sharper frequency selectivity than High-SR fibers. The results of the simulation support this framework; Low-SR ANFs were shown to have sharper frequency tuning (Figure 1B) than High-SR ANFs throughout a range of characteristic frequencies (CFs). While Low-SR ANFs have sharper frequency selectivity (Figure 1B), High-SR ANFs have finer temporal resolution, as the rate of change of the mean firing rate in High-SR ANFs was well above that of Low-SR fibers in the simulation (Figure 1C). It would seem that Low-SR and Medium-SR fibers (i.e. Lower-SR fibers) are optimized for “place theory” frequency coding and High-SR fibers are optimized for “volley-theory” synchronous phase locking. Future modeling efforts might maintain the integrity of these two parallel pathways, optimized for fine spectral (Lower-SR) and fine temporal (High-SR) resolution, by separating rather than summing their respective outputs.
